# Risk Management in students using the FMEA Method (Prospective Approach) to identify ‎risky cases in term of suicide and its prevention 

**Published:** 2019-08

**Authors:** Haniyeh Rezaei, Tayyebeh Rezaei

**Affiliations:** ^*a*^Tabriz University of Medical Sciences, Tabriz, Iran.

## Abstract

**Background:**

Risk management refers to the process of adopting policies to accept, identify, evaluate, ‎control, minimize or reduce the assessed risks. Risk management is described in five activities: ‎communicating and counseling, creating background, risk assessment, Monitoring and review. ‎Among these five activities, risk assessment is the most complex one and it includes ‎identifying, analyzing and evaluating risk. Risk Assessment Method, FMEA (Failure Mode and ‎Effects Analysis) was first presented by the US Army for the aerospace industry in 1960s; it was ‎also considered as the basis for managing pre-incident crisis management issues in healthcare ‎systems. Its benefits include anticipating and preventing problems, reducing costs, shortening ‎the time to improve quality, and achieving secure and highly reliable processes. Considering the ‎increasing rate of suicide and its adverse effects on family and society, a prospective approach ‎can be employed for psychological improvement, and evaluate risky cases before the event, and ‎take the necessary interventions to reduce the risk and the likelihood of committing suicide.‎

**Methods:**

This is a research project in the form of safety prospective approach on vulnerable groups. In ‎this project, it is recommended to clinic psychologists at health centers to use a standard ‎questionnaire on probability of suicide in a scheduled program for all high schools in Marand ‎city and assess the students psychologically and evaluate the probability of having suicidal ‎thoughts and finally identify risky students. In the next step, counseling and psychology sessions ‎will be held for identified students; then involved, predisposing factors and inhibitors will be ‎identified in these students. Then, according to the score gained from assessment, students will ‎be prioritized and individual psychology sessions will be held in order to accurately assess the ‎problems of these students and enhance their defense mechanisms. Also, in order to improve the ‎ability to cope with the stress and crisis, some educational sessions will be held for the families ‎of these students and appropriate psychological strategies for proper communication with ‎children, increasing self-esteem, reducing family pressures and ... can be provided for them.‎

**Results:**

In order to implement this plan, a session should be held for all the psychologists of the center, ‎and the objectives of the meeting should be explained to them, and then the action plan must be ‎developed in the form of a Gantt chart. In the next step, the prioritization of schools must be ‎done according to specific areas with high suicide rates. The necessary coordination should also ‎be provided with schools. All data must be used for accurate assessments of managers and ‎teachers. Also, after t identification in improving the mental health of students, teachers can ‎play a very useful role. In this regard, the training teachers seems necessary considering the ‎purposes.‎

**Conclusions:**

Given the importance of a prospective approach in safety, FMEA method is a suitable way for ‎identifying and prioritizing the risks of the society, especially schools. By managing these risks ‎and planning properly, the possible risks can be managed at the time of happening. Also, ‎holding life skills workshops, training adaptive stress skills, training to control stress and ‎anxiety, and using spiritual issues can be helpful to improve the mental health of vulnerable ‎people and to manage possible psychological risks.‎

**Keywords:**

Psychological safety, FMEA, Suicide probability, Students

